# Cumulative Incidence of All-Cause Knee Injury, Concussion, and Stress Fracture among Transgender Patients on Gender-Affirming Hormone Therapy: An Exploratory Retrospective Cohort Study

**DOI:** 10.3390/ijerph20227060

**Published:** 2023-11-13

**Authors:** Emily W. Miro, Eliza Taylor, Andrew Curtin, Michael G. Newman, Dominik Ose, Jordan Knox

**Affiliations:** Division of Family Medicine, Department of Family and Preventive Medicine, University of Utah, Salt Lake City, UT 84108, USA

**Keywords:** transgender, knee injury, concussion, stress fracture

## Abstract

Previous research has shown a discrepancy in incidences of knee injuries, stress fractures, and concussions between cisgender men and women. Little is known regarding the incidence of musculoskeletal injuries among patients on gender-affirming hormone therapy (GAHT). This retrospective cohort study examines cumulative incidence of knee injuries, concussions, and stress fracture injuries among transgender patients on GAHT at one health system from 2011–2020. Using relevant ICD-9 and 10 codes, incidences of knee injury, concussion, and stress fracture were calculated. Cohorts included 1971 transgender and 3964 cisgender patients. Transgender patients had significantly higher incidence of all-cause knee injuries over the study period, 109 (5.5%) versus 175 (4.4%) (*p* < 0.001; OR: 2.14, 95% CI [1.17–3.92]). Subgroup analysis showed significantly higher incidence of knee injuries among cisgender men (5.6%) versus cisgender women (4.1%) (*p* = 0.042) and among transgender women (6.6%) versus cisgender women (4.1%) (*p* = 0.005). There were no significant differences between incidences of concussion and stress fracture between groups. This sample showed that patients on GAHT had increased cumulative incidences of all-cause knee injury compared to controls but similar cumulative incidences of concussion and bone-stress injuries. Transgender women on exogenous estrogen had significantly higher cumulative incidences of all-cause knee injuries compared to cisgender women.

## 1. Introduction

Transgender and gender-diverse individuals are those whose gender identities differ from the sex assigned to them at birth [1]. Transgender women were assigned to the male sex at birth but identify as female. Transgender men were assigned to the female sex at birth but identify as male. Over 1.6 million adults identify as transgender in the United States. Of those, approximately 38.5% identify as transgender women, 35.9% as transgender men, and 25.6% as gender nonconforming [2].

Gender-affirming medical care is deeply personal and individualized and may include a combination of factors, including, but not limited to, psychological support, gender-affirming hormone therapy (GAHT), and surgical intervention. GAHT typically includes a combination of estrogen and anti-androgenic medications among transgender women and testosterone therapy among transgender men [1].

The initiation of GAHT has been shown to reduce gender dysphoria and positively impact quality of life [3]. Editorials and recent studies recognize the short-term effects of GAHT on lean body mass, muscular strength, hemoglobin, and hematocrit and long-term effects of GAHT on bone mineral density [4,5,6,7,8]. However, the short-term and long-term effects of GAHT on musculoskeletal health have otherwise not been elucidated.

While conversation surrounding the allowance and regulation of transgender athletes in elite sport has gained international attention recently, there is a notable paucity of data describing the incidence of injuries among these patients who have unique risk factors, including long-term exposure to exogenous hormones [9,10,11].

The effects of some endogenous hormones on injury risk, however, have been extensively studied in cisgender individuals. Previous studies have reported increased incidences of anterior cruciate ligament (ACL) injuries [12,13,14], concussions [15,16,17], and stress fractures in cisgender women compared to cisgender men [18,19,20,21,22]. It has been theorized that endogenous estrogen and testosterone may contribute to difference in injury incidence [13,14,15,21,22], and particularly compelling publications suggest that estrogen increases susceptibility of the ACL to injury [14,23,24,25,26,27,28,29]. Conversely, additional studies examining incidence of both all-cause and sports-related knee injuries, not limited to ACL sprains, show higher incidences of knee injuries in cisgender men compared to cisgender women [30,31].

This study aims to provide preliminary data on incidence of all-cause knee injuries, concussions, and stress fractures among a transgender patient population prescribed GAHT.

## 2. Materials and Methods

### 2.1. Design and Population

This exploratory retrospective cohort study was conducted at the University of Utah Health system in Salt Lake City, Utah. Available data were collected from 2011 through 2020. University of Utah Health is Utah’s only academic health care system and provides various forms of transgender health services, including GAHT and gender-affirming surgery. No patients were directly involved with this study. The University of Utah Institutional Review Board (IRB 00145187) exempted the study. An a priori power analysis was not performed before this exploratory study.

### 2.2. Transgender Cohort Selection

Using a previously described approach [32], transgender patients on GAHT were included in the transgender cohort if they met the following criteria: (1) Alive and between 18 and 65 years old at the time of an included medical system encounter, (2) Specific diagnoses recorded at the encounter OR gender data fields indicated transgender status (Table 1), and (3) the patient chart listed a prescription for hormone therapy anytime between 2011 and 2020. Consent was waived for this study.

### 2.3. Cisgender Cohort Selection

A cohort of transgender patients was matched with cisgender patients in a 1:2 ratio using a nonreplacement selection algorithm. A 1:2 ratio was chosen due to the significant difference in sample size of transgender patients within our health system compared to cisgender patients. Eligible cisgender patients were randomly selected from a pool of patients who were living and had at least one encounter in the health system. Cisgender patients were matched to transgender patients if they met the following criteria: (1) There was a +/− 1 year difference in age between the transgender and cisgender patients, (2) There was a +/− 1 year time difference between the most recent patient encounters within the health system. In this random selection, there was no recruitment process that could lead to a self-selection bias.

From the matched dataset, patients were excluded if they had prescriptions for both masculinizing and feminizing GAHT. Ultimately, 3964 cisgender or non-binary patients formed the matched cohort, whereas 885 transgender women, 928 transgender men, and 158 non-binary/other patients were included in subsequent clinical and demographic characteristic analyses.

### 2.4. Variables

From these records, three musculoskeletal injury categories were identified by ICD codes: Knee injury, Concussion, and Stress fracture. Further details of ICD codes are available in Table S1.

### 2.5. Statistical Analysis

Frequencies (n) and relative frequencies (%) were used to describe the characteristics of groups of patients for all categorical variables. *p*-values were calculated by chi-square test. The chi-square tests were conducted to assess the associations between the two cohorts. Further chi-square tests were conducted for musculoskeletal injury groups to assess associations with cisgender men, cisgender women, transgender men, and transgender women. There were also unadjusted calculations made of crude odds ratio (OR) and 95% confidence interval (95% CI).

*p*-value significance level was set to 0.05, such that a *p*-value less than or equal to the significance level suggested a significant association between the compared variable groups. The mean and standard deviation were calculated for continuous variables (age and body mass index (BMI)). All the above analyses were conducted in RStudio R version 4.2.0.

## 3. Results

### 3.1. Clinical and Demographic Characteristics

There were 5939 participants in our study: 1971 (33.2%) transgender patients and 3964 (66.8%) cisgender patients (Table 2). Among all transgender patients, 45.2% identified as transgender women, 46.8% as transgender men, and 8% as nonbinary (Table 2). Of all cisgender patients, 54.2% were female, 45.7% were male, and 0.1% identified as nonbinary. The average age of participants was 32 (Standard Deviation (SD) = 11). Among all subjects, 73.3% identified as White, 2.9% as Asian, and 2.2% as Black or African American. Mean BMI was 27.9 (SD = 7.41). Musculoskeletal injuries were identified in 230 controls and 125 cases (Table 3). Further demographic data for this subgroup is shown in Table 3.

### 3.2. Gender-Affirming Hormone Therapy

Among the transgender patient cohort, 46.9% were on feminizing hormones, 51.1% were on masculinizing hormones, and 1.9% were on a combination of feminizing and masculinizing hormones. GAHT medications by gender identity are described in Table 4. In this sample, 77% of transgender women were on a combination of estrogen and spironolactone; 10% were on estrogen alone; and 8% were on a combination of estrogen, spironolactone, finasteride, and progesterone. In this sample, 5% were on spironolactone alone. All transgender men were on testosterone-only GAHT (Table 4).

### 3.3. Knee Injury

Among the total study population, 284 (4.8%) participants were found to have had a knee injury over the study time period. Transgender patients had significantly higher incidence of knee injuries compared to cisgender patients (5.5% versus 4.4%, *p* < 0.001; OR: 2.14, 95% CI [1.17–3.92]) (Table 2).

All knee injury data were further analyzed to examine incidence in cisgender women and men and transgender women and men. Subgroup analysis showed significantly higher cumulative incidences of knee injuries among cisgender men (5.6%) versus cisgender women (4.1%) (*p* = 0.042; OR:0.73, 95% CI [0.55–0.98]) (Table 5). Analysis also showed significantly higher cumulative incidence of knee injuries among transgender women (6.6%) versus cisgender women (4.1%) (*p* = 0.005; OR:1.64, 95% CI [1.17–2.31]) (Table 5). There was no significant difference in cumulative incidence of knee injuries among cisgender men versus transgender men or cisgender women versus transgender men.

### 3.4. Concussion

There was no significant difference observed between incidences of concussions in transgender patients versus cisgender patients. Only 38 (0.7%) subjects had a diagnosis of concussion during the study period, 7 (0.4%) in the transgender cohort, and 31 (0.8%) in the cisgender cohort (*p* = 0.090) (Table 2). Concussion injury data by gender subgroup are shown in Table 5. Subgroup analysis did not find a significant difference in incidences between cisgender men versus transgender women. Further subgroup analysis was unable to be performed due to sparse cells and low sample size (Table 2).

### 3.5. Stress Fracture

Additionally, no significant difference was observed between cumulative incidence of stress fracture in transgender patients versus cisgender patients. Over the study period, 12 (0.2%) participants had a diagnosis of stress fracture, 3 (0.2%) in the transgender cohort, and 9 (0.2%) in the cisgender cohort (*p* = 0.643) (Table 2). Stress fracture data by gender subgroup is shown in Table 5. Subgroup analysis was unable to be performed due to sparse cells (Table 5).

## 4. Discussion

To our knowledge, this study is the first to examine cumulative incidence of a select group of common, all-cause musculoskeletal injuries in transgender patients. The all-cause and sport-related incidence of common musculoskeletal injuries has been extensively studied in cisgender individuals.

### 4.1. Knee Injury

Knee injuries are a common occurrence, and hundreds of studies report the incidence of both sport-related and all-cause knee injuries between cisgender males and females.

We found that transgender patients on GAHT had a significantly higher cumulative incidence of all-cause knee injuries compared to cisgender patients within our health system. These data were further analyzed for possible differences in incidence among patients with exposure to exogenous versus endogenous hormones. We found that transgender women receiving exogenous estrogen and/or testosterone-blocking agents had significantly higher incidence of knee injury compared to cisgender women.

This study cannot conclude that this is due to the influence of GAHT alone, in large part due to its retrospective design. There are likely multiple factors at play, not the least of which could be the significantly higher BMI in the study’s population of transgender patients. Whether this variable is independent or secondary to GAHT, as well as its correlation with knee injury risk, must be considered further. In essence, this comparison of transgender women on GAHT (male skeletal structure with exogenous female hormones) to cisgender women (female skeletal structure with endogenous female hormones) might indicate that exogenous female hormones are a greater risk factor for knee injury. One might argue that this comparison aligns with the cisgender men > cisgender women rates of knee injuries. When we analyzed the incidence of all-cause knee injuries between cisgender men (male skeletal structure with male hormones) and transgender women (male skeletal structure with exogenous female hormones), we found no significant difference in cumulative incidence in our study population.

Without any comparative studies available, we can only use these results to theorize that the hormonal influence of exogenous sex hormones may increase the risk of knee injuries—perhaps at a molecular, structural level or perhaps through its influence on BMI.

Analysis of all-cause knee injuries between cisgender men and women in our sample revealed a higher incidence of knee injuries in cisgender men compared to cisgender women, which was consistent with previous reports of sports-related and all-cause knee injuries [30,31].

### 4.2. Concussion and Stress Fracture

Discrepancy in incidences of concussions and stress fractures between cisgender male and female athletes has been previously reported and theorized to stem, at least in part, from hormonal influence [15,16,17,18,19,20,21,22]. We performed similar analysis on incidence of concussions and bone stress injuries in our transgender and cisgender cohorts. We found a low incidence of concussion and bone stress injuries in both cohorts. We found no significant difference in injury rates between groups.

### 4.3. Limitations

This study includes many notable limitations. While the sample size is comparatively large, small injury sample size limited the power of our statistical analysis. *A priori* power analysis was not performed before the start of this study. We hope that future, multi-institution studies may allow for a larger sample of transgender patients and increased statistical power. Given our low sample size, we were unable to draw conclusions regarding differences in incidence of concussion and stress fractures between cohorts.

We acknowledge that BMI plays a pivotal role in knee injury and likely significantly influences our results. Indeed, BMI data show a higher percentage of overweight and obese transgender patients compared to controls, which may account for differences in incidence of knee injury between groups. BMI data were not available for a portion of our sample, and thus results were not adjusted for BMI. Race, ethnicity, and socioeconomic status may also have significant implications on incidence of knee injury, concussion, and stress fracture. A regression analysis was not performed, as in doing so we would be ignoring an imperative bias by the unmeasured confounders. Future study on incidence of these common musculoskeletal injuries should adjust for these variables and provide further clarity on the influence of specific variables on overall knee injury data.

Furthermore, our study is notably limited by lack of data on previous injury, physical activity level, sports participation, and profession, which can influence incidence of musculoskeletal injury. This information was unable to be obtained via the retrospective chart review. Future prospective research is necessary to obtain information on level of physical activity in patients and sports participation in patients to further inform musculoskeletal injury patterns.

While all patients in this study have been seen within our hospital system in the past 10 years, we acknowledge that they have varied exposure to GAHT. We did not analyze musculoskeletal injury with regard to duration of exposure to GAHT or level of measured estradiol or testosterone at time of injury. The timing of injury and its association with hormone levels could not be determined in this preliminary, retrospective design. Future studies should aim to further elucidate these relationships.

Classification of transgender cases into “sex assigned at birth” and “gender identity” relied, in part, on patient reporting of these data. Self-reported “sex assigned at birth” and “gender identity” data were incomplete in many of our transgender case records (620 records). If self-reported gender identity was unavailable, gender identity was determined by GAHT medications prescribed and verified by reported sex and sex assigned at birth. If sex assigned at birth was unavailable, it was determined by reported gender identity and GAHT medications.

While the authors have experience in the field of transgender medicine, they are not experts on formulations of exogenous hormones and may have made a clinical error in the interpretation of various formulations and combinations of estrogen, testosterone, and additional hormones used in GAHT. We attempted to utilize inclusive and accurate language in composing this report and apologize if there are any errors.

## 5. Conclusions

This exploratory retrospective cohort study provides preliminary data on incidence of select all-cause musculoskeletal injuries among transgender patients. The authors found that patients on GAHT had significantly increased cumulative incidence of knee injuries compared to an age-matched cisgender control cohort. Furthermore, transgender female patients on feminizing therapy had significantly increased incidence of knee injuries compared to the cisgender female sample. We did not find any significant difference in knee injury among patients on exogenous versus endogenous testosterone. We found no significant differences in incidences of concussion or bone stress injuries among our transgender patients and controls and were unable to further analyze this data due to sparse cells and unrecorded data.

We are unable to conclude that exposure to GAHT is directly related to increased cumulative incidence of knee injuries compared to cisgender controls. Rather, the intent of this study is merely to report incidence of select musculoskeletal complaints in the transgender population compared to cisgender controls. Many unmeasured confounding variables exist in these populations, and we are unable to account for these variables with a regression given our sample size. Further study is warranted to develop more detailed descriptions of the incidence of musculoskeletal injuries in the transgender population as a whole as well as the transgender athlete population. These studies must include larger samples to increase power in these analyses and provide clarity regarding the influence of specific confounding variables, including, but not limited to BMI, race, ethnicity, age, level of activity, socioeconomic status, and exposure to GAHT.

While many previous studies have reported discrepancies in incidence of sport related injuries between cisgender men and women, there is a paucity of data surrounding injury incidence in transgender individuals. As more international attention turns to regulating the participation of transgender athletes in sport, we aim to elucidate the musculoskeletal risks to these patients compared to their cisgender counterparts. Further characterization of injury incidences among transgender athletes will allow us to provide equitable healthcare to this vulnerable population.

## Figures and Tables

**Table 1:** Transgender Patient on Gender-Affirming Hormone Therapy Criteria; Diagnoses at Encounter or Gender Data Field.

Sex Assigned at Birth ^a^	Sex/Gender Identity at Encounter	Transgender Patient Status (with Corresponding Gender-Affirming Hormone Therapy)
Male	Female	Transgender Woman
Female	Male	Transgender Man
-	Transgender Woman	Transgender Woman
-	Transgender Man	Transgender Man
-	Non-Binary	Non-binary/Other/Unknown
-	Other	Non-binary/Other/Unknown
-	Agender	Non-binary/Other/Unknown
-	Gender Queer	Non-binary/Other/Unknown
-	Gender X	Non-binary/Other/Unknown
-	-/Choose not to Disclose	Non-binary/Other/Unknown

^a^ Sex defined as sex assigned at birth. If sex assigned at birth data unavailable, then determined as reported sex and verified by gender identity. Gender identity determined by reported gender identity. If unavailable, then determined by GAHT medications taken and by reported sex and sex assigned at birth. Gender identity of Transgender Man also categorized by testosterone-only GAHT medication records. Transgender on GAHT criteria were defined by the following: (1) Specific diagnoses recorded at the encounter OR gender data fields indicated transgender status (Table 1) AND (2) the patient chart listed a prescription for hormone therapy anytime between 2011 and 2020.

**Table 2:** Clinical and Demographic Characteristics.

	Population		Transgender Cohort		Cisgender Cohort		*p*-Value
	N	%	N	Vert %	N	Vert %	
Included in Analysis (horiz %)	5935	100	1971	33.2	3964	66.8	
Musculoskeletal Injury							0.096
Knee	284	4.8	109	5.5	175	4.4	<0.001 *
Concussion	38	0.7	7	0.4	31	0.8	0.090
Stress Fracture	12	0.2	3	0.2	9	0.2	0.643
Multiple	21	0.4	6	0.3	15	0.4	0.647
Sex ^a^							<0.001 *
Female	3186	53.7	1036	52.6	2150	54.2	
Male	925	15.6	923	46.8	1812	45.7	
Unknown/Non-Binary	14	0.2	12	0.6	2	0.1	
Gender Identity							
Transgender Woman	-	-	890	45.2	-	-	
Transgender Man	-	-	923	46.8	-	-	
Non-binary/Other/Unknown	-	-	158	8.0	-	-	
GAHT Medications	-	-			-	-	
Feminine Affirming ^b^	-	-	925	46.9	-	-	
Masculine Affirming ^b^	-	-	1008	51.1	-	-	
Mix			38	1.9			
Age, mean (Standard Deviation)	32	(11)	32	(11)	32	(11)	
Age-Categorized							0.999
18 to 34 years	4165	70.2	1383	70.2	2782	70.2	
35 to 49 years	1189	20.0	395	20.0	794	20.0	
50 years and older	581	9.8	193	9.8	388	9.8	
Ethnicity							<0.001
Non-Hispanic/Latino	4555	76.7	1671	84.8	2880	72.7	
Hispanic/Latino	769	13.0	199	10.1	570	14.4	
Unknown	611	10.3	97	4.9	514	13.0	
Race							<0.001
White or Caucasian	4353	73.3	1645	83.5	2708	68.3	
American Indian/Alaska Native	52	0.9	25	1.3	27	0.7	
Asian	173	2.9	45	2.3	128	3.2	
Black or African American	129	2.2	41	2.1	88	2.2	
Native Hawaiian/Pacific Islander	73	1.2	17	0.9	56	1.4	
Other	621	10.5	135	6.8	486	12.3	
Unknown	534	9.0	63	3.2	471	11.9	
BMI, mean (Standard Deviation)	27.99	(7.41)	28.50	(7.10)	27.63	(7.07)	
Obesity-categorized (kg/m^2^)							<0.001
Underweight (<18.50)	129	2.2	55	2.8	74	1.9	
Healthy Weight (18.50–24.99)	1688	28.4	681	34.6	1007	25.4	
Overweight (25.00–29.99)	1206	20.3	501	25.4	705	17.8	
Class 1 obesity (30.00–34.99)	723	12.2	315	16.0	408	10.3	
Class 2 obesity (35.00–39.99)	383	6.5	197	10.0	186	4.7	
Class 3 obesity (40.00+)	303	5.1	148	7.5	155	3.9	
Unknown ^c^	1503	25.3	74	3.8	1429	36.0	

GAHT: Gender-Affirming Hormone Therapy. BMI: body mass index. * There was a significant difference in musculoskeletal injuries being knee injuries between transgender patients and cisgender patients (*p* < 0.001; crude OR: 2.14, 95% CI [1.17–3.92]). There was also a significant difference in biological sex between transgender patients and cisgender patients (*p* < 0.001), with higher number of unknown/nonbinary in transgender patients. *p*-value by chi-square test, ^a^ Sex defined as sex assigned at birth. If sex assigned at birth data unavailable, then determined as reported sex and verified by gender identity. Gender identity determined by reported gender identity. If unavailable, then determined by GAHT medications taken and by reported sex and sex assigned at birth. Transgender on GAHT criteria were defined by the following: (1) Specific diagnoses recorded at the encounter OR gender data fields indicated transgender status (Table 1) AND (2) the patient chart listed a prescription for hormone therapy anytime between 2011 and 2020. ^b^ Included GAHT Medication(s) feminizing hormones: (Estradiol), (Spironolactone), (Estradiol Valerate; Spironolactone), (Estradiol; Estradiol Cypionate; Estradiol Valerate; Finasteride; Spironolactone), (Estradiol; Estradiol Cypionate; Estradiol Valerate; Spironolactone), (Estradiol; Estradiol Valerate; Finasteride; Spironolactone), (Estradiol; Estradiol Valerate; Spironolactone), (Estradiol; Finasteride; Spironolactone), (Estradiol; Spironolactone), (Estradiol; Estradiol Cypionate; Spironolactone), (Estradiol; Estradiol Valerate; Finasteride; Progesterone Micronized; Spironolactone). Included GAHT Medication(s) masculinizing hormones: (Testosterone Cypionate), (Testosterone Cypionate; Testosterone Enanthate), (Testosterone; Testosterone Cypionate), (Testosterone Cypionate and Prop (Propionate)), (Testosterone; Testosterone Cypionate; Testosterone Enanthate), and (Testosterone Undecanoate). Feminine affirming are Estradiol or Spironolactone-type formulations; Masculine affirming are Testosterone-type formulations. Records, including both Feminine-affirming and Masculine-affirming hormones, are included in the mixed category (estradiol type + testosterone type). ^c^ 74 case records had no BMI record.

**Table 3:** Musculoskeletal Injury subpopulation clinical and demographic characteristics.

		Sub Population	Case		Control		*p*-Value
	N	%	N	Vert %	N	Vert %	
Included in Analysis (horiz %)	355	100	125	35	230	65	
Age, mean (Standard Deviation)	36	(13)	38	(12)	35	(13)	
Age-Categorized							0.010
18 to 34 years	198	56	55	44	143	62	
35 to 49 years	99	28	45	36	54	23	
50 years and older	58	16	25	20	33	14	
Sex ^a^							0.295
Female	172	48	64	51	108	47	
Male	183	52	61	49	122	53	
Gender Identity							
Transgender Woman	-	-	64	51	-	-	
Transgender Man	-	-	40	32	-	-	
Non-binary	-	-	8	6	-	-	
Other	-	-	11	9	-	-	
Choose not to disclose	-	-	2	2	-	-	
Ethnicity							0.923
Non-Hispanic/Latino	302	85	105	84	197	86	
Hispanic/Latino	33	9	12	10	21	9	
Unknown	20	6	8	6	12	5	
Race							0.395
White or Caucasian	285	80	101	81	180	78	
American Indian/Alaska Native	5	1	4	3	1		
Asian	7	2	2	2	5	2	
Black or African American	11	3	4	3	7	3	
Native Hawaiian/Pacific Islander	8	2	2	2	6	2	
Other	29	8	9	7	20	9	
Unknown	14	4	3	24	11	5	
BMI, mean (Standard Deviation)	28.60	(8.44)	29.49	(8.87)	28.04	(8.13)	
Obesity-categorized (kg/m^2^)							0.246
Underweight (<18.50)	5	1	-	-	5	2	
Healthy Weight (18.50–24.99)	134	38	44	35	90	39	
Overweight (25.00–29.99)	73	21	32	25	42	18	
Class 1 obesity (30.00–34.99)	54	15	20	16	33	15	
Class 2 obesity (35.00–39.99)	35	10	15	12	20	9	
Class 3 obesity (40.00+)	30	8	14	11	16	7	
Unknown	24	7	-	-	24	10	

*p*-value by chi-square test or Fisher’s exact test as applicable. ^a^ Sex defined as sex assigned at birth. If sex assigned at birth data unavailable, then determined as reported sex and verified by gender identity. Gender identity determined by reported gender identity. If unavailable, then determined by GAHT medications taken and by reported sex and sex assigned at birth. Transgender on GAHT criteria were defined by the following: (1) Specific diagnoses recorded at the encounter OR gender data fields indicated transgender status (Table 1) AND (2) the patient chart listed a prescription for hormone therapy anytime between 2011 and 2020.

**Table 4:** Gender-Affirming Hormone Therapy Medication by Gender Identity.

	Case (Vertical %)			Total (Vert %)
	Transgender Women	Transgender Men	Other Patient (Non-Binary)	
Estrogen-only formulations ^a^	92 (10)	-	8 (5)	100 (5)
Testosterone-only formulations	-	923 (100)	79 (50)	1002 (51)
Spironolactone	48 (5)	-	26 (16)	74 (4)
Estrogen formulation; Spironolactone	681 (77)	-	37 (23)	718 (36)
Estrogen formulation; Other ^b^	69 (8)	-	8 (5)	77 (4)
Total (Horiz. %)	890 (45)	923 (47)	158 (8)	1971

^a^ Estrogen Formulations; Testosterone Formulations excluded (four cases). ^b^ “Other” formulation Finasteride (n = 6), finasteride-included mixes (n = 69), and Progesterone Micronized-included mixes (n = 5).

**Table 5:** Musculoskeletal Injuries Incidence Comparison by Gender.

	Transgender Women	Transgender Men	Cisgender Men	Cisgender Women		*p*-Values
Population ^a^	890	923	1812	2150		
Knee Injury	59 (6.6)	38 (4.1)	101 (5.6)	89 (4.1)	Cisgender Men vs. Transgender Women	0.315 (OR:1.20, 95% CI [0.86–1.68])
					Cisgender Women vs. Transgender Men	0.977 (OR:0.99, 95% CI [0.67–1.47])
					Cisgender Women vs. Transgender Women	0.005 (OR:1.64, 95% CI [1.17–2.31])
					Cisgender Men vs. Cisgender Women	0.042 (OR:0.73, 95% CI [0.55–0.98])
Concussion	6	3	21	21	Cisgender Men vs. Transgender Women	0.241 (OR:0.58, 95% CI [0.23–1.44])
					Cisgender Women vs. Transgender Men	N/A
					Cisgender Women vs. Transgender Women	N/A
					Cisgender Men vs. Cisgender Women	0.5769 (OR:0.84, 95% CI [0.46–1.55])
Stress Fracture	4	1	8	5	Cisgender Men vs. Transgender Women	N/A
					Cisgender Women vs. Transgender Men	N/A
					Cisgender Women vs. Transgender Women	N/A
					Cisgender Men vs. Cisgender Women	N/A

*p*-value by chi-square test, as applicable. Calculated unadjusted odds ratio (OR) and 95% confidence interval (95% CI). ^a^ Estrogen Formulations; Testosterone Formulations excluded (four cases).

## Data Availability

The data presented in this study are available on request from the corresponding author. The data are not publicly available due to privacy.

## Supplementary Materials

The following supporting information can be downloaded at: https://www.mdpi.com/article/10.3390/ijerph20227060/s1, Table S1: Included Musculoskeletal Injuries Diagnosis at Encounter.

## References

[1] Coleman E., Radix A.E., Bouman W.P., Brown G.R., de Vries A.L.C., Deutsch M.B., Ettner R., Fraser L., Goodman M., Green J. (2022). Standards of Care for the Health of Transgender and Gender Diverse People, Version 8. Int. J. Transgender Health.

[2] Herman J.L., Flores A.R., O’neill K.K. (2022). How Many Adults and Youth Identify as Transgender in the United States?.

[3] Skewis L.F., Bretherton I., Leemaqz S.Y., Zajac J.D., Cheung A.S. (2021). Short-Term Effects of Gender-Affirming Hormone Therapy on Dysphoria and Quality of Life in Transgender Individuals: A Prospective Controlled Study. Front. Endocrinol..

[4] Marra J., Law A.M.Y., Conlon E. (2021). Clinical Considerations in Caring for Transgender Athletes. Am. Fam. Physician.

[5] Harper J., O’Donnell E., Khorashad B.S., McDermott H., Witcomb G.L. (2021). How does hormone transition in transgender women change body composition, muscle strength and haemoglobin? Systematic review with a focus on the implications for sport participation. Br. J. Sports Med..

[6] Delgado-Ruiz R., Swanson P., Romanos G. (2019). Systematic Review of the Long-Term Effects of Transgender Hormone Therapy on Bone Markers and Bone Mineral Density and Their Potential Effects in Implant Therapy. J. Clin. Med..

[7] Fighera T.M., Ziegelmann P.K., da Silva T.R., Spritzer P.M. (2019). Bone Mass Effects of Cross-Sex Hormone Therapy in Transgender People: Updated Systematic Review and Meta-Analysis. J. Endocr. Soc..

[8] Roberts T.A., Smalley J., Ahrendt D. (2021). Effect of gender affirming hormones on athletic performance in transwomen and transmen: Implications for sporting organisations and legislators. Br. J. Sports Med..

[9] Harper J., Hirschberg A.L., Jose M., Ritzén M., Vilain E., Taylor J., Riley L., Mitchell R., Elwani R., Mohamed-Ali V. (2015). IOC Consensus Meeting on Sex Reassignment and Hyper-and Rogenism.

[10] Ingram B.J., Thomas C.L. (2019). Transgender Policy in Sport, A Review of Current Policy and Commentary of the Challenges of Policy Creation. Optom. Vis. Sci..

[11] World Athletics. IAAF Introduces New Eligibility Regulations for Female Classification|PRESS-RELEASE|Quai Antoine, Monaco. https://worldathletics.org/news/press-release/eligibility-regulations-for-female-classifica.

[12] Montalvo A.M., Schneider D.K., Yut L., Webster K.E., Beynnon B., Kocher M.S., Myer G.D. (2019). “What’s my risk of sustaining an ACL injury while playing sports?” A systematic review with meta-analysis. Br. J. Sports Med..

[13] Hewett T.E., Zazulak B.T., Myer G.D. (2007). Effects of the Menstrual Cycle on Anterior Cruciate Ligament Injury Risk: A Systematic Review. Am. J. Sports Med..

[14] Chidi-Ogbolu N., Baar K. (2019). Effect of Estrogen on Musculoskeletal Performance and Injury Risk. Front. Physiol..

[15] Covassin T., Swanik C.B., Sachs M.L. (2003). Sex Differences and the Incidence of Concussions among Collegiate Athletes. J. Athl. Train.

[16] Broshek D.K., Kaushik T., Freeman J.R., Erlanger D., Webbe F., Barth J.T. (2005). Sex differences in outcome following sports-related concussion. J. Neurosurg..

[17] Colvin A.C., Mullen J., Lovell M.R., West R.V., Collins M.W., Groh M. (2009). The Role of Concussion History and Gender in Recovery from Soccer-Related Concussion. Am. J. Sports Med..

[18] Changstrom B.G., Brou L., Khodaee M., Braund C., Comstock R.D. (2015). Epidemiology of Stress Fracture Injuries among US High School Athletes, 2005–2006 through 2012–2013. Am. J. Sports Med..

[19] Rizzone K.H., Ackerman K.E., Roos K.G., Dompier T.P., Kerr Z.Y. (2017). The Epidemiology of Stress Fractures in Collegiate Student-Athletes, 2004–2005 through 2013–2014 Academic Years. J. Athl. Train..

[20] Khosla S., Monroe D.G. (2018). Regulation of Bone Metabolism by Sex Steroids. Cold Spring Harb. Perspect. Med..

[21] Nattiv A., De Souza M.J., Koltun K.J., Misra M., Kussman A., Williams N.I., Fredericson M. (2021). The Male Athlete Triad-A Consensus Statement from the Female and Male Athlete Triad Coalition Part 1: Definition and Scientific Basis. Clin. J. Sport Med..

[22] Nattiv A., Loucks A.B., Manore M.M., Sanborn C.F., Sundgot-Borgen J., Warren M.P. (2007). The Female Athlete Triad. Med. Sci. Sports Exerc..

[23] Parsons J.L., Coen S.E., Bekker S. (2021). Anterior cruciate ligament injury: Towards a gendered environmental approach. Br. J. Sports Med..

[24] Liu S.H., Al-Shaikh R.A., Panossian V., Finerman G.A.M., Lane J.M. (1997). Estrogen Affects the Cellular Metabolism of the Anterior Cruciate Ligament: A Potential Explanation for Female Athletic Injury. Am. J. Sports Med..

[25] Yu W.D., Panossian V., Hatch J.D., Liu S.H., Finerman G.A.M. (2001). Combined Effects of Estrogen and Progesterone on the Anterior Cruciate Ligament. Clin. Orthop. Relat. Res..

[26] Konopka J.A., DeBaun M.R., Chang W., Dragoo J.L. (2016). The Intracellular Effect of Relaxin on Female Anterior Cruciate Ligament Cells. Am. J. Sports Med..

[27] Lee C.A., Lee-Barthel A., Marquino L., Sandoval N., Marcotte G.R., Baar K. (2015). Estrogen inhibits lysyl oxidase and decreases mechanical function in engineered ligaments. J. Appl. Physiol..

[28] Adachi N., Nawata K., Maeta M., Kurozawa Y. (2008). Relationship of the menstrual cycle phase to anterior cruciate ligament injuries in teenaged female athletes. Arch. Orthop. Trauma Surg..

[29] Herzberg S.D., Motu’apuaka M.L., Lambert W., Fu R., Brady J., Guise J.-M. (2017). The Effect of Menstrual Cycle and Contraceptives on ACL Injuries and Laxity: A Systematic Review and Meta-Analysis. Orthop. J. Sports Med..

[30] Gray A.M., Buford W.L. (2015). Incidence of Patients With Knee Strain and Sprain Occurring at Sports or Recreation Venues and Presenting to United States Emergency Departments. J. Athl. Train..

[31] Yawn B.P., Amadio P., Harmsen W.S., Hill J., Ilstrup D., Gabriel S. (2000). Isolated Acute Knee Injuries in the General Population. J. Trauma.

[32] Proctor K., Haffer S.C., Hodge C., James C.V., Lichtenstein M., Stein L., Connolly E., Goldstein Z.G., Martinson T., Tiersten L. (2016). Identifying the Transgender Population in the Medicare Program. Transgender Health.

