# Supplementary material for: Cumulative Incidence of All-Cause Knee Injury, Concussion, and Stress Fracture among Transgender Patients on Gender-Affirming Hormone Therapy: An Exploratory Retrospective Cohort Study
Source: Int J Environ Res Public Health. 2023 Nov 13;20(22):7060. doi: 10.3390/ijerph20227060 (PMC10671107; doi:10.3390/ijerph20227060)
Supplement: Supplementary file 1 [file ijerph-20-07060-s001.zip › ijerph-2596730-supplementary.pdf]

Supplemental Materials

Table S1: Included Musculoskeletal Injuries Diagnosis at Encounter

**Musculoskeletal Injury**	**ICD Code (Category Codes)[a]**
**Knee**	719.06<br>719.46<br>844.9<br>848.9<br>959.7<br>M23.8X<br>R93.6<br>S83.511A<br>S83.511D<br>S83.512A<br>S89.80XA<br>S89.90XA<br>S89.90XD<br>S89.91XA<br>S89.91XD<br>S89.92XA<br>S89.92XS
**Concussion**	850.0<br>850.11<br>850.5<br>850.9<br>S06.0X9A
**Stress Fracture**	733.94<br>733.95<br>805.4<br>M84.352A<br>M84.361D<br>M84.375A<br>M84.371A; M84.374A; M84.374D; M84.374G; M84.377G; M84.378G; Z87.312

ICD: International Classification of Diseases

ICD codes were checked for exclusions (Category codes) *Z98.890, Z87.828, Z47.89, F07.81, T84.XA*.
